# The perspective of socially vulnerable youth on healthcare services: a Portuguese cross-sectional study

**DOI:** 10.1186/s13690-025-01680-7

**Published:** 2025-08-01

**Authors:** Carlos Franclim Silva, Viviana Barreira, Daniel Beirão, Luísa Sá, Paulo Santos

**Affiliations:** 1 MEDCIDS– Department of Medicine of Community, Information and Health Decision Sciences, Faculty of Medicine, University of Porto, Porto, Portugal; 2 CINTESIS@RISE, MEDCIDS, Faculty of Medicine, University of Porto, Porto, Portugal; 3 ULSM– Local Health Unit of Matosinhos (USF Horizonte), Matosinhos, Portugal

**Keywords:** Adolescent, Young adult, Social vulnerability, Patient-centeredness, Youth-friendly, Community health, Primary healthcare facilities

## Abstract

Socially vulnerable adolescents are often forgotten in the healthcare system, which is more prone to manage those who are assigned to the system, leaving others. We aimed to characterise the perception of these patients about preferences and priorities concerning healthcare services in their medical appointments, relevant healthcare topics, and value attributed to the contact with their physicians.

This cross-sectional study surveyed a group of adolescents living in institutional settings to enhance a patient-centred approach and, therefore, higher health promotion and better health outcomes for adolescents and young adults.

A total of 571 adolescents with a mean age of 17 (55.4% females) answered the survey. Primary healthcare centres were the main location for medical surveillance. The most wanted topics were diet (83%), diseases (82%), and exercise (75%), with less emphasis surrounding topics such as free time, tobacco, and drugs. A significant association was found between valuing physicians’ characteristics and perceived doctor skills.

This research highlights the pertinence of understanding the priorities and preferences of institutionalised socially vulnerable adolescents to reinforce equity and create a cosy environment for everyone.

**Table:** 

**Text box 1. Contributions to the literature**
• Vulnerable adolescents and young adults predominantly use public Health centres for medical services, including lifestyle management.
• Factors influencing appointment preferences include physician characteristics, location, schedule, privacy, waiting list, and fees.
• Preferences for health topics align with public health priorities, except for tobacco and alcoholic beverage consumption.
• Physician characteristics and skills are key factors in appointment preferences, especially in discussing sexuality, framed by perceived competency.

## Introduction

Adolescence is a stage of rapid growth, and the underlying greater autonomy establishes a distinctive set of challenges and exploration. Adolescents have a heightened susceptibility to social evaluation and to adopt risk behaviour; they are also endowed with plasticity that enables adaptive social learning, both a vulnerability and an opportunity [1, 2]. Young adults are typically defined as persons aged between 18 and 24 [3]. They are a critical target for policies regarding preventable health problems [4]. 

Socially disadvantaged people are at greater risk of adverse social and environmental conditions, exposure, and consequences on their health and well-being [5–7]. Adverse experiences, such as dysfunctional family dynamics, poverty, discrimination, or abuse, may lead to poor health outcomes, such as psychoactive substance misuse, sexual and reproductive problems, violent injuries, and diminished life opportunities [8–12]. Additionally, healthy environments and family and school connectedness are protective of several health outcomes, both in youth and adulthood [13, 14]. Portuguese law provides special protection to young people at risk. Since 1999, Law 147/99 has defined the situations in which entities with legal authority can intervene to protect children whose safety, health, education, development or upbringing are at risk, directly or indirectly. This protection is guaranteed to children up to 18 and maintained until their socioeconomic stability. According to the Portuguese Social Security Institution report, 28% of children and young people at critical social risk show behaviour problems, like aggression, opposition, defiance, or rule-breaking, 26% have a significant disease or disability, and 8% state illicit substance misuse [15]. Social vulnerability is associated with lower access to healthcare services [16]. Therefore, including socially vulnerable youth in research and health policies is critical.

The behavioural model of health services embraces contextual characteristics, individual features, health behaviours, and significant outcomes, such as consumer satisfaction and evaluated and perceived health [17]. The sociodemographic characteristics, beliefs, health policies, financing, and organisation are significant determinants [17]. According to the World Health Organisation, adolescent-friendly services should be equitable, accessible, appropriate, effective, and acceptable, meeting adolescents’ expectations, ensuring privacy and confidentiality, and delivering staff with proper characteristics and competency [18]. In Portugal, the national health program for children and youth establishes the protocol for children’s assessment according to key ages, specifically determined to critical steps for development, healthy lifestyles promotion, adherence to preventive measures such as vaccination or contraception, prevention of risky behaviours, and screening for difficulties at a social level, including situations of increased risk. The most common indicators for youth-friendly sexual and reproductive health services are accessibility (easy access, affordable or free of charge, reasonable waiting times), staff characteristics, competency (being welcoming, friendly, respectful, non-judgmental, trustworthy, providing adequate information, and involving patients in decisions), confidentiality and privacy [19–21]. Proper communication in a patient-provider relationship is crucial; adolescents value a non-judgmental and straightforward approach and appreciate understanding the purpose of risk assessment, which balances the potential sense of invasion intrinsic to the health interview [22, 23]. Additionally, health providers should assess the patient’s developmental stage and prior experiences to suit the provided information.

Family physicians, encompassing health promotion and preventive competencies, the first level of youth access to the healthcare system, tend to assign low priority to young people [24]. Youth do not consider healthcare professionals to be the leading counsellors regarding health topics, and they feel more comfortable discussing health topics with family, friends, and partners [25]. A North American and a Brazilian survey showed that this age group seeks less health care for prevention or health recovery [17, 26]. 

Primary healthcare services present distinctive forms of organisations worldwide that attend to conceptual domains: vision, resources, structure, and practices [27]. Despite some complementary private services, the base of Portuguese medical assistance is provided by the public National Health System, based on a Beveridge model, funded mainly by the government budget. Primary healthcare covers the whole country through a network of local health centres, guaranteeing access, continuity of care, comprehensiveness, and advocacy for all populations. The 2006 reform introduced the payment for performance for primary care health centres. Only the units that achieve a pre-specified set of quality indicators and their providers, including doctors, nurses and administrative staff, are paid by the incentive system (B-model). Children have always been free from any charge in public health centres. Since 2020, this exemption has been extended to all Primary Care services, allowing true accessibility regardless of financial impairment. Access to healthcare facilities is free for every patient, irrespective of age, who can schedule their appointments independently, through a tutor, other relative solicitation or provider suggestion. Also, it is possible to have an appointment without a previous schedule by direct request in a daily open consultation.

The main topics and issues to address or consider in a medical surveillance consultation to manage a specific problem of an adolescent or young adult are education or employment, home, peer-group activities, literacy, psychoactive substance use and misuse, sleeping, diet, physical activity, emotions, depression and anxiety, sexuality, intimate partner violence, safety, and immunisation [28–47]. It is critical to integrate target-population expectations in a medical appointment. However, significant strategies regarding health promotion should be considered.

Service utilisation can be assessed from the patient’s or the physician’s perspectives [48]. The present research opted to explore the patient’s view. This research aims to characterise socially vulnerable adolescents and young adults regarding healthcare services, their preferences regarding facilities, communication, and the main topics to address. This study may enhance a patient-centred approach and provide higher health promotion and better health outcomes for adolescents and young adults.

## Methods

A cross-sectional study was conducted, with analytic intention, on Portuguese youth between 14 and 24 years of age living at shelter homes in northern Portugal through a self-administered written form survey from February/2020 to June/2020.

### Participants and sampling

The Portuguese Social Security Institution states that 7553 children and young people are identified as at critical social risk [15]. Portugal’s mainland has 212 social shelter homes for hosting children and young people at social risk, according to Portuguese Ministry of Labour and Social Security data [6]. The north of Portugal corresponds to about one-third of the total population, encompassing the districts of Aveiro, Braga, Bragança, Guarda, Porto, Vila Real, Viana do Castelo and Viseu, including 91 shelter homes. All institutionalised adolescents and young adults aged 14 to 24 from northern Portugal were eligible to participate (*n* = 1920).

### Data collection

The participants were invited by the representatives of social institutions, who agreed to cooperate under the General Data Protection Regulation rules. Those who did not speak or understand the Portuguese language and those without the physical or mental ability to fill out the questionnaire were excluded.

### Questionnaire and variables

Adolescents’ and young people’s perspectives towards health care services were evaluated.

The questionnaire incorporated different dimensions such as social and demographic characteristics (age, gender, academic status, birthplace), usual medical service used (HC, public hospital, private service, does not usually use health services or other); usual reason for a medical appointment (health surveillance, health problems, obtaining medical certificates or other); priorities concerning healthcare services through a 5-point Likert scale (confidentiality, saving information shared during the appointment; privacy before, during and after the appointment; physicians’ characteristics and qualities; comfort; waiting list; convenient opening time; administrative procedures, namely scheduling process; distance to appointment location; service fees); priorities concerning healthcare appointment topics through a 5-point Likert scale (diet; diseases; exercise; sexually transmitted diseases; contraception; sleep; sexuality; education and occupation; home environment; emotions; tobacco; drugs; free time); patient-doctor communication priorities through a 5-point Likert scale (feeling welcomed by the physician; being understood by the physician; physician explains health problems and therapeutic plans; being involved in decision-making; trust built with the physician; physician knowledge and training); general service facilities preferences regarding a medical appointment for which the participant ordered the dimensions considering the importance attributed to each one, with the first corresponding to the most crucial dimension and fourth to the least important (doctor’s characteristics; location; timetable and medical appointment fee), attributing a positive score to the first preference shown. The 5-point Likert scale measured variables were categorised as positive cases, and a score of 4 or 5 was given. Regarding the sex and gender approach, we used Heidari’s recommended definitions [49]. 

### Ethical issues

Participants (or their guardians) signed the informed consent form. Questionnaires were sealed in individual envelopes after being filled out, ensuring anonymity.

### Statistical analysis

We used descriptive statistics to calculate prevalence and the modified Wald method to determine the 95% confidence intervals (CI). The inferential analysis used Student’s t-tests, nonparametric tests, and the Chi-square or Fisher test, depending on the variables. Normal distribution was tested using the Kolmogorov–Smirnov test. Multivariate analysis used a binary logistic regression model to estimate the relationship between factors and dependent variables. The significance level was set at 0.05. Data were encoded and registered in a Microsoft Office Excel 2010 database and analysed using IBM SPSS Statistics, version 28.0 (IBM Corp., Armonk, NY, USA).

## Results

The total sample included 571 participants (55.4% females, mean age of 17 ± 2.2 years old, 32% aged 18+), representing an estimated answer rate of 36% after adjusting the eligible population dimension to those in the institutions who accepted to participate. All 91 institutions included in the official data were contacted: 41 institutions accepted enrolment in the study, 31 institutions did not answer the call after at least three contacts, seven institutions declined due to inactivity at that time, six declined due to not presenting participation criteria, and four institutions declined for other reasons.

Most were born in Portugal (94.7%, *n* = 531). Table 1 shows the sociodemographic characteristics. Health surveillance was the main reason for visiting the doctor (*n* = 269; 50.3%), followed by the need to manage a medical condition (45.2%). HC was the usual health service used for a medical appointment, 65.4% (*n* = 332). Regarding preferences related to medical appointments, participants consider doctor skills the main factor, 34%. Concerning the importance of the approach in a medical appointment, the highest scoring topics were diet, 83%, diseases, 82%, and exercise, 75%.

**Table 1:** Sociodemographic characteristics, habits, and preferences of population

		*n*	Proportion (%)
**Age**	Years, mean ± SD	17.0 ± 2.2-	-
	At least 18 years	180	32
**Gender**	Female	313	55
	Male	252	45
	Other	0	-
**Place of birth (country)**	Portugal	531	95
	Other	30	5
**Education** (**last attended year)**	Primary	348	64
	Secondary	189	35
	Higher	8	1
	Secondary or Higher	197	36
**Usual healthcare service**	Local Health Centre	332	65
	Public hospital	128	25
	Private service	10	2
	Does not use	36	7
	Other	2	0
**Motive for a medical appointment**	Health surveillance	269	50
	Health problems	242	45
	Medical certificate	9	2
	Other	15	3
**Medical appointment topics scored as important**	Diet	456	83
	Diseases	443	82
	Exercise	419	75
	Sexually transmitted disease	394	73
	Contraception	391	72
	Sleep	377	69
	Sexuality	368	68
	Education and occupation	369	67
	Home	332	63
	Emotions	343	63
	Tobacco	291	54
	Drugs	282	53
	Free time	280	51
	Alcohol	273	51
**General service facilities’ preferences for a medical appointment**	Doctor skills	173	34
	Location	140	28
	Timetable	123	24
	Fee	73	14

Abbreviations: OR, Odds Ratio; CI, Confidence Interval

Multiple logistic regression was used to analyse the determinants of the outcomes valuing doctor skills, location, timetable, and the fee of a medical appointment (Table 2). A significant association was found between valuing physicians’ characteristics and competency and valuing doctors’ skills (OR = 3.83, 95%CI: 1.61–9.11, *p* = 0.002). There is a significant association between the distance to the appointment site and the valuing of the factor location (OR = 4.39, 95%CI: 1.61–11.99, *p* = 0.004). The outcome variable timetable presents a significant association with the variable’s privacy before, during and after the appointment (OR = 4.63, 95%CI: 1.53–14.07, *p* = 0.007), waiting list (OR = 0.43, 95%CI: 0.22–0.83, *p* = 0.012), convenient opening hours, and timetable (OR = 2.05, 95%CI: 1.03–4.09, *p* = 0.041), and the physicians’ characteristics and competency (OR = 0.46, 95%CI: 0.21- 1.00, *p* = 0.05). Valuing the appointment fee presents an association with the waiting list (OR = 2.11, 95%CI: 1.02–4.35, *p* = 0.043) and the service fee (OR = 2.16, 95%CI: 1.19–3.91, *p* = 0.011). Investigation outcomes were extended to other determinants through a multiple logistic regression analysis, as seen in Table 3. Valuing the sexuality topic in a medical appointment is associated with valuing the doctor’s skills (OR = 2.03, 95%CI: 1.13–3.63, *p* = 0.017). A significant association exists between using an HC and valuing timetable (OR = 0.63, 95%CI: 0.39- 1.00, *p* = 0.045).

**Table 2:** Multivariate analysis of the determinants of the outcomes valuing the characteristics of Doctor skills and service facilities for a medical appointment

		Service facilities for a medical appointment		
	**Doctor skills**	**Location**	**Timetable**	**Fee**
	OR (95% CI)	OR (95% CI)	OR (95% CI)	OR (95% CI)
**Health service facilities**				
Confidentiality, saving information shared during the appointment	0.95 (0.41–2.21)	1.25 (0.39–4.06)	0.50 (0.21–1.18)	1.88 (0.74–4.79)
Privacy, before, during and after the appointment	0.43 (0.16–1.14)	1.65 (0.41–6.72)	4.63* (1.53–14.07)	0.47 (0.18–1.19)
Physicians’ characteristics and qualities	3.83* (1.61–9.11)	0.59 (0.22–1.56)	0.46** (0.21-1.00)	0.81 (0.38–1.75)
Comfort	1.14 (0.59–2.19)	0.82 (0.33–2.05)	0.82 (0.41–1.61)	1.00 (0.51–1.97)
Waiting list	1.28 (0.68–2.42)	0.97 (0.40–2.37)	0.43* (0.22–0.83)	2.11* (1.02–4.35)
Convenient opening hours, timetable	0.56 (0.31–1.01)	1.17 (0.50–2.76)	2.05* (1.03–4.09)	0.83 (0.45–1.55)
Distance	0.67 (0.37–1.19)	4.39* (1.61–11.99)	0.95(0.49–1.82)	0.73 (0.39–1.36)
Administrative procedures, Scheduling process	0.77 (0.44–1.33)	1.90 (0.86–4.18)	1.31 (0.70–2.44)	0.64 (0.35–1.17)
Service fee	1.13 (0.65–1.96)	0.45(0.21–0.96)	0.63 (0.34–1.16)	2.16* (1.19–3.91)
**Patient-doctor communication**				
Feeling welcomed by the physician	0.68 (0.19–2.38)	0.65 (0.16–2.62)	1.55 (0.45–5.31)	1.23 (0.36–4.19)
Being understood by the physician	1.76 (0.50–6.12)	2.70 (0.54–13.57)	0.60 (0.19–1.90)	0.56 (0.18–1.72)
The physician explains health problems and therapeutic plans	1.58 (0.58–4.35)	0.56 (0.70 − 0.21)	0.72 (0.28–1.85)	0.98 (0.37–2.62)
Be involved in decisions	0.65 (1.19–0.56)	0.66 (0.25–1.77)	1.55 (0.67–3.59)	0.76 (0.36–1.60)
The build-up of trust with the physician	0.99 (0.40–2.44)	0.49 (0.16–1.47)	1.46 (0.57–3.80)	1.24 (0.51–3.05)
The physician’s knowledge and training	2.04 (0.91–4.61)	1.31 (0.48–3.60)	0.38* (0.18–0.83)	1.28 (0.54–3.01)

Abbreviations: OR, Odds Ratio; CI, Confidence Interval, *significance level, *p* < 0.05, ** *p* = 0.05

**Table 3:** Multivariate analysis of patients’ characteristics associated to a medical appointment related to Doctor skills and service facilities

		Service facilities, medical appointment		
	**Doctor skills**	**Location**	**Timetable**	**Fee**
	OR (95% CI)	OR (95% CI)	OR (95% CI)	OR (95% CI)
**Age group (at least 18 years old)**	1.08 (0.60–1.94)	1.20 (0.54–2.71)	0.69 (0.36–1.31)	1.18 (0.61–2.28)
**Gender (male)**	1.23 (0.80–1.91)	0.66 (0.35–1.23)	0.86 (0.54–1.37)	1.22 (0.75-2.00)
**Education (at least attended secondary)**	1.19 (0.66–2.12)	0.89 (0.40-2.00)	0.81 (0.44–1.52)	1.10 (0.57–2.10)
**Usual health service (Local health centre)**	1.43 (0.90–2.27)	1.05 (0.55–1.98)	0.63* (0.39-1.00)	1.15 (0.68–1.94)
**Motive for a medical appointment (surveillance)**	1.12 (0.97–1.29)	0.81 (0.58–1.13)	0.98 (0.83–1.14)	0.97 (0.82–1.15)
**Important topic (sexuality)**	2.03* (1.13–3.63)	0.50 (0.24–1.05)	1.15 (0.64–2.06)	0.59 (0.32–1.10)
**Important topic (sexually transmitted disease)**	0.67 (0.33–1.36)	1.15 (0.44–3.04)	1.01 (0.48–2.11)	1.58 (0.69–3.63)
**Important topic (contraception)**	0.99 (0.51–1.93)	1.11 (0.44–2.84)	1.02 (0.51–2.06)	0.86 (0.41–1.81)
**Important topic (emotions)**	0.74 (0.47–1.19)	0.89 (0.47–1.71)	1.25 (0.76–2.06)	1.14 (0.67–1.94)
**Important topic (drugs)**	1.02 (0.61–1.69)	0.78 (0.39–1.56)	0.90 (0.53–1.52)	(0.74–2.34) 1.31

Abbreviations: OR, Odds Ratio; CI, Confidence Interval, * *p* < 0.05

## Discussion

Most vulnerable adolescents and young adults use an HC as their leading health service. About one-half access health services to carry out medical surveillance, giving importance to discussing the following topics: diet, diseases, and physical exercise. Physician skills are the main factor regarding medical appointment preferences, especially if participants value the subject of sexuality. In attending to vulnerable youth perceptions, significant congruence between physicians’ characteristics and competency and physicians’ skills was found, as well as the distance to the location and the location itself. The schedule variable is congruent with the appointment’s characteristics, such as opening hours and timetable. Still, it is also related to other determinants, such as privacy, waiting list, and the physicians’ characteristics and competency. Congruent results concerning the appointment fee and a significant association with the waiting list variable were found. Participants who valued mostly the available timetable for a medical appointment referred to a lesser use of HC services.

It must be stated that data collection took place during the COVID-19 pandemic debut in Portugal, which may have impacted both participants’ enrolment and their perceptions regarding health services. Nevertheless, we obtained a response of 36%, which may be underestimated, given that 31 institutions did not answer the recruitment call, and some could be inactive or not fulfil the participation criteria. Institutions were obtained by consulting official social assistance services records, which include updated information, making it improbable that eligible institutions were misidentified or not contacted, taking into account usual government procedures allowing for reliable information availability. Although we did not use a validated questionnaire, we found significant congruence between relevant concepts, such as medical skills, location, timetable, and service fees, that enhanced our results and conclusions.

The attendance of HC as the usual medical service was expected, given their characteristics (suitable, coordinated by the government, spread across the country and free of charge) when considering the social vulnerability in the studied population.

A Norwegian survey showed concordant data that young people usually contact their general physician [24]. High demand for health services due to general surveillance fits this age group’s general morbidity and mortality attributes [1–4]. Social vulnerability contributes to additional healthcare needs and healthcare service access difficulties [15, 16, 26]. No relevant studies were found to compare and disclose our results regarding the usual reason to visit the doctor.

The topics targeting a medical appointment approach were scored as important by at least one-half of the participants, corroborating previous literature [28–47]. A preference was shown concerning diet, diseases, and physical exercise topics versus tobacco, drugs, or free time. In this age group, the main risk factors for non-communicable diseases, the leading cause of death worldwide, are physical inactivity, insufficient fruit or vegetable intake, and carbonated soft drink consumption [50, 51]; therefore, participants’ expectations fit public health priorities. Additionally, tobacco and alcohol use among adolescents are still frequent risk factors with the highest potential economic return associated with preventive interventions, disagreeing with the participants’ priorities [50, 51]. These results highlight the need to balance the youth’s and health providers’ agendas to enhance health determinants. Free time is a broad concept, allowing for misunderstanding regarding its pertinence within the scope of a medical appointment. Further investigation may extend the characterisation of participants’ priorities concerning the topics approached in a medical appointment, disclosing their concerns and problems and providing healthcare professionals or other counsellors with information regarding needs and expectations.

We found distinct approach expectation patterns concerning the topics of sexuality, sexually transmitted diseases, contraception, and emotions in medical appointments. The sexuality topic expectation was the only topic with a significant association with the outcome variable, medical skills preferences. The sexuality approach should embrace many dimensions, such as promoting well-being, beyond preventing significant adverse outcomes [52, 53]. These results strengthen the broad scope of the sexuality concept from the youth’s perspective and their varying expectations and preferences in a medical appointment, valuing the doctor’s skills substantially.

Valuing timetables presented a significant positive association with valuing privacy and convenient opening hours, and a negative association with valuing the waiting list and physicians’ characteristics and competency. Participants who valued the timetable of a medical appointment more referred to the most minor use of HC services. The privacy variable presents a more substantial, predictable value than the variable opening hour, and the timetable is not expected. Further investigation should explore this finding to understand the concept of timetables and schedules for youth and additional significant factors, such as occupational or personal obligations or availability of institutional staff, parents, or guardians accompanying the adolescents and young adults to a medical appointment.

Valuing the appointment fee positively correlates with the determinant of valuing the waiting list. Community model services tend to value continuity more than accessibility and attribute less importance to financial return, in opposition to some alternative organisational models, where accessibility and financial return are priorities [27]. HC, the participants’ preferred service, presents properties of the community service model that may explain the association of valuing the waiting list as an accessibility concern indicator and the possible need to use a paid service to face HC limitations.

## Conclusion

Most vulnerable adolescents and young adults use HC, a public community model service. About one-half access health services to carry out medical surveillance, considering important discussion topics such as diet, diseases, and physical exercise. The doctor’s skills are the main factor regarding medical appointment preferences, especially if participants value sexuality topics in a broad scope, including contraception, emotions, behaviours and sexually transmitted diseases.

The current study provides valuable insights into the healthcare priorities and preferences of socially vulnerable adolescents living in institutions. These findings highlight the importance of understanding this population’s specific preferences and needs to tailor health-related services effectively.

In the future, targeted interventions and policies should be developed to address the specific healthcare needs identified in this study, focusing on promoting health literacy, fostering healthy behaviours, and reducing healthcare disparities among socially vulnerable adolescents.

## Data Availability

Database is deposited in the server of University of Porto and available under justified request.
